# Supplementary material for: Combination of chemotherapy and PD-1 blockade induces T cell responses to tumor non-mutated neoantigens
Source: Commun Biol. 2020 Feb 25;3:85. doi: 10.1038/s42003-020-0811-x (PMC7042341; doi:10.1038/s42003-020-0811-x)
Supplement: Supplementary file 1 — Supplementary Information [file 42003_2020_811_MOESM1_ESM.pdf]

Supplementary Figures

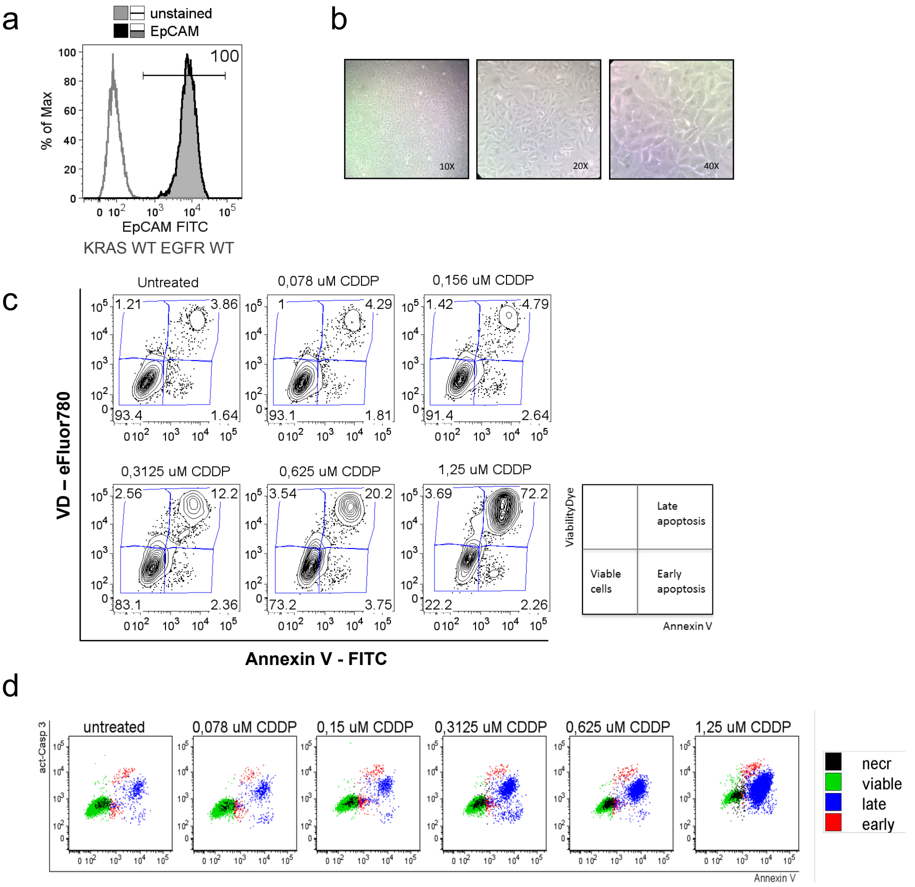

Supplementary Figure 1

Supplementary Figure 1. Characterization of primary NSCLC cell line and of CDDP-dependent apoptosis requirements. (a) Flow cytometry analysis of primary NSCLC cell line derived from a LUAD patient. Purity of tumor cells was confirmed by the specific marker of epithelial cell adhesion molecule (EpCAM). Histogram reported % of Max of EpCAM positive cells. (b) Morphology of NSCLC cell line growing in complete medium (10X, 20X, 40X magnification). (c) Representative flow cytometry analysis of apoptotic assay in which NSCLC cell line were treated for 72 h with increased concentration of CDDP. Viable, late or early apoptotic fractions were identified using Viability Die (VD) and Annexin V. (d) Flow cytometry analysis of apoptotic assay (previously described). After CDDP treatment, viable (green), early (red) or late apoptosis (blue) and necrotic (black) cells were identified

according to activated (act)-Caspase 3 and Annexin V expression. 0,625 μM of CDDP for 72 h was selected as best condition of treatment.

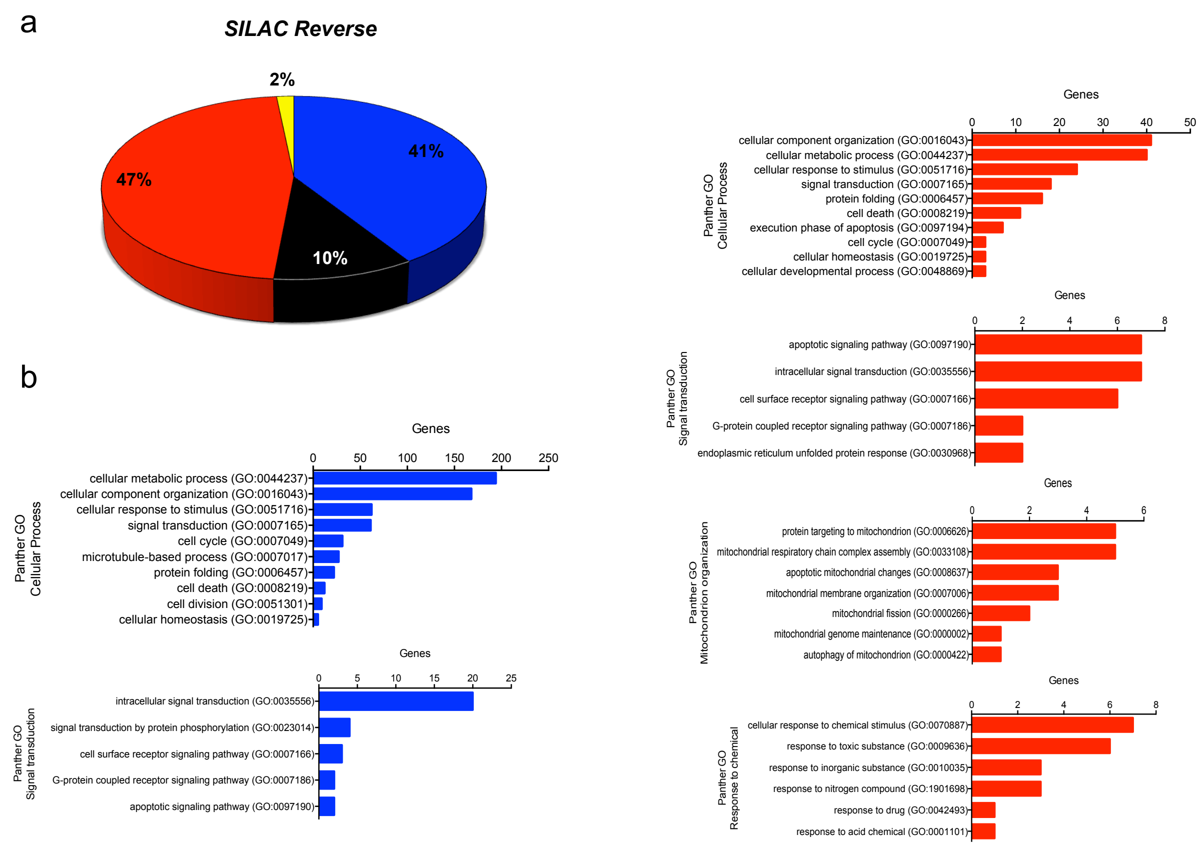

Supplementary Figure 2

Supplementary Figure 2. SILAC reverse experiments and Biological function analysis. (a) Pie chart describing the CDDP-induced proteomic changes in SILAC reverse experiment. Upregulated proteins (abundance changes >2-fold increase) are displayed in red; downregulated in blue (abundance changes <2-fold increase); no change in protein abundance in black. Protein fragments are showed in yellow. (b) Biological process analysis was performed on both downregulated (blue bars) and upregulated (red bars) proteins. Indicated categories were classified according to Gene Ontology database. All analyses were performed with PANTHER classification system. Number of enriched genes was reported for each category in all graphs.

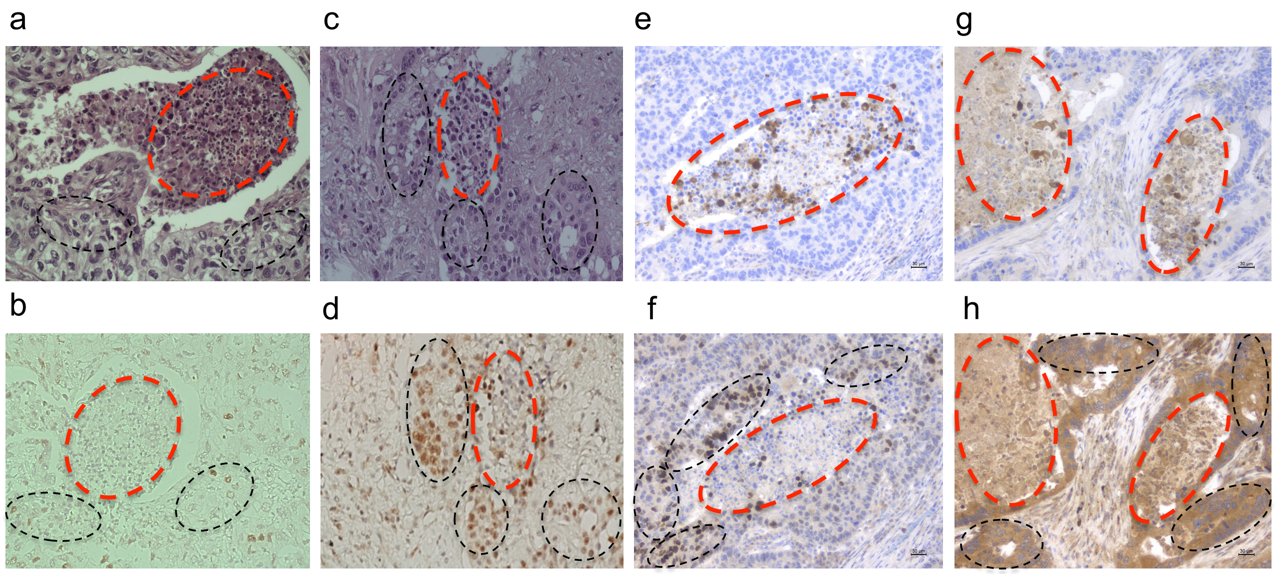

Supplementary Figure 3

Supplementary Figure 3. Immunohistochemical analysis in NSCLC tissues. (a-d) Serial hematoxylin-eosin (HE) stained sections of NSCLC from patients without (a, b) or upon chemotherapy (c, d) (magnification 20x); (a, c) black and red dashed circle delimits representative areas containing live and apoptotic tumor cells, respectively: apoptotic tumor cells (single or in clusters) are characterized by nuclear condensation and dense eosinophilic cytoplasm; (b, d) serial HE sections were stained with anti-PSAP mAb: black and red dashed circles delimit representative areas containing PSAP positive or negative live and apoptotic tumor cells, respectively. (e-h) Serial formalin fixed paraffin-embedded cancer tissue sections NSCLC samples from patients upon chemotherapy were stained with the anti-Cleaved Caspase-3 antibody (e, g), mAb anti-PSAP (f) or polyclonal anti-LYRIC (h) (magnification 20x); (e) serial cancer sections were stained with anti-Cleaved Caspase-3: red dashed circles delimit representative areas containing Cleaved Caspase-3

positive (apoptotic) or negative tumor cells; (f) sections were stained with anti-PSAP mAb: black and red dashed circles delimit representative areas containing PSAP positive or negative live and apoptotic tumor cells, respectively; (g) serial cancer sections were stained with anti-Cleaved Caspase-3: red dashed circles delimit representative areas containing Cleaved Caspase-3 positive (apoptotic) or negative tumor cells; (h) sections were stained with anti-LYRIC: black and red dashed circles delimit representative areas containing LYRIC positive or negative live and apoptotic tumor cells, respectively.

a

Protein Name	Peptide Number	Sequence
PROACTIVATOR POLYPEPTIDE	1	LDRNLEKNSTKQEILAALEK
	2	STKQEILAALEKGCSFLPDP
	3	ALEKGCSFLPDPYQKQCDQF
	4	LPDPYQKQCDQFVAEYEPVL
	5	QCDQFVAEYEPVLIEILVEV
OLFACTORY RECEPTOR 5H2	6	VFYTIIIPLLNPIIYSLRNK
	7	LLNPIIYSLRNKQVIDSFTK
	8	YSLRNKQVIDSFTKMVKRNV
PROTEIN LYRIC	9	MAARSWQDELAQQAEEGSAR
	10	ELAQQAEEGSARLREMLSVG
	11	GSARLREMLSVGLGFLRTEL
	12	LREMLSVGLGFLRTELGLDL
RAS AND EF-HAND DOMAIN CONTAINING PROTEIN	13	LQLWDTAGQERFRSIAKSYF
	14	QERFRSIAKSYFRKADGVLL
	15	FRSIAKSYFRKADGVLLLYD
ZINC TRANSPORTER SLC39A7	16	EEEKETRGVQKRRGGSTVPK
	17	VQKRRGGSTVPKDGPVRPQN
	18	TVPKDGPVRPQNAEEEKRGL
	19	RPQNAEEEKRGLDLRVSGYL
	20	KRGLDLRVSGYLNLAADLAH
	21	GLDLRVSGYLNLAADLAHNF
ADP/ATP TRANSLOCASE 2	22	QITADKQYKGIIDCVVRIPK
	23	KGIIDCVVRIPKEQGVLSFW
	24	RIPKEQGVLSFWRGNLANVI
	25	LSFWRGNLANVIRYFPTQAL
	26	GNLANVIRYFPTQALNFAFK
CATHEPSIN D	27	FTVVFDTGSSNLWVPSIHCK
	28	SSNLWVPSIHCKLLDIACWI
	29	IHCKLLDIACWIHHKYNSDK
	30	ACWIHHKYNSDKSSTYVKNG
	31	YNSDKSSTYVKNGTSFDIHY
RUV B -LIKE 2	32	RIGAHSHIRGLGLDDALEPR
	33	RGLGLDDALEPRQASQGMVG
	34	LEPRQASQGMVGQLAARRAA
	35	GMVGQLAARRAAGVVLEMIR
	36	RRAAGVVLEMIREGKIAGRA
	37	RAAGVVLEMIREGKIAGRAV

b

POOL	7	8	9	10	11	12
1	1	2	3	4	5	6
2	7	8	9	10	11	12
3	13	14	15	16	17	18
4	19	20	21	22	23	24
5	25	26	27	28	29	30
6	31	32	33	34	35	36
						37

Supplementary Figure 4

Supplementary Figure 4. Overlapping peptides derived from NSCLC-associated NM-neoAgs and matrix scheme. (a) Custom library of 37 overlapping peptides derived from 8 selected proteins, identified as upregulated in apoptotic cell fraction. Each peptide shows a 20 amino acid sequence with overlapping region of 12 residues. A sequential number was assigned for each peptide and a distinct color code was assigned for each protein. Protein name and relative amino acid sequences were showed. (b) Matrix scheme of 37 overlapping peptides mixed in 12 pools (horizontal lines number 1-6 and vertical columns number 7-12) in which each peptide was shared between two pools.

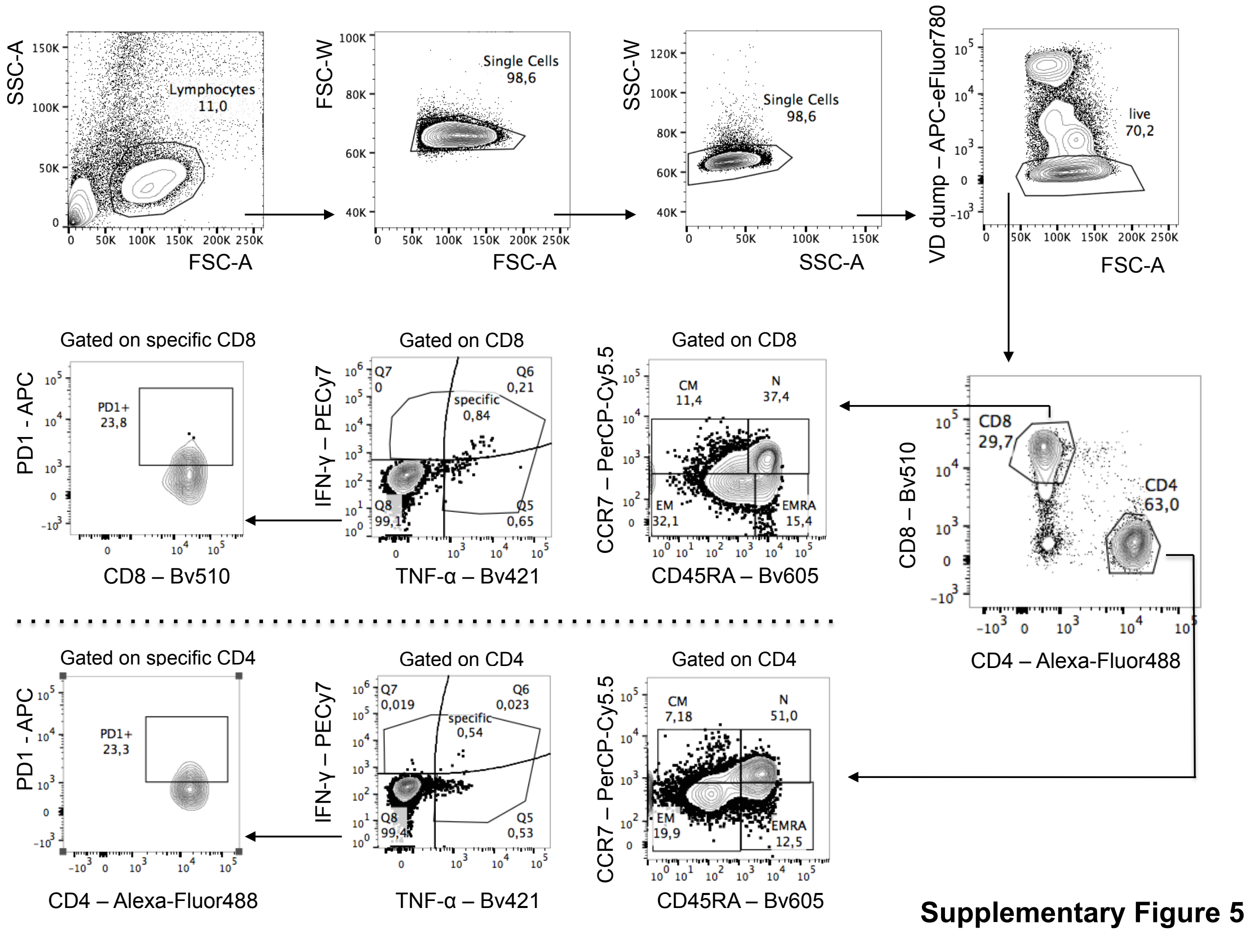

Supplementary Figure 5

Supplementary Figure 5. Gating strategy procedure. A representative NSCLC patient treated with CDDP is shown. Lymphocytes were first gated by the physical parameter Forward and Side scatter area (FSC-A and SSC-A). Doublets and debris were excluded by plotting the width (W) against the area of FSC and SSC parameters. Dead cells were excluded using viability dye (VD), and $CD8^+$ and $CD4^+$ T cells were gated into live cells. Dump channel was included for excluding B cells, monocytes and NK cells (CD19, CD14, CD16, CD56). Within both $CD8^+$ and $CD4^+$ T cells populations, N, CM, EM, EMRA subsets, as well as IFN-γ and/or TNF-α producing cells in response to peptide pools (specific $CD8^+$ or $CD4^+$ T cells) were gated. PD-1^+ cells were gated on specific $CD8^+$ or $CD4^+$ T cells.

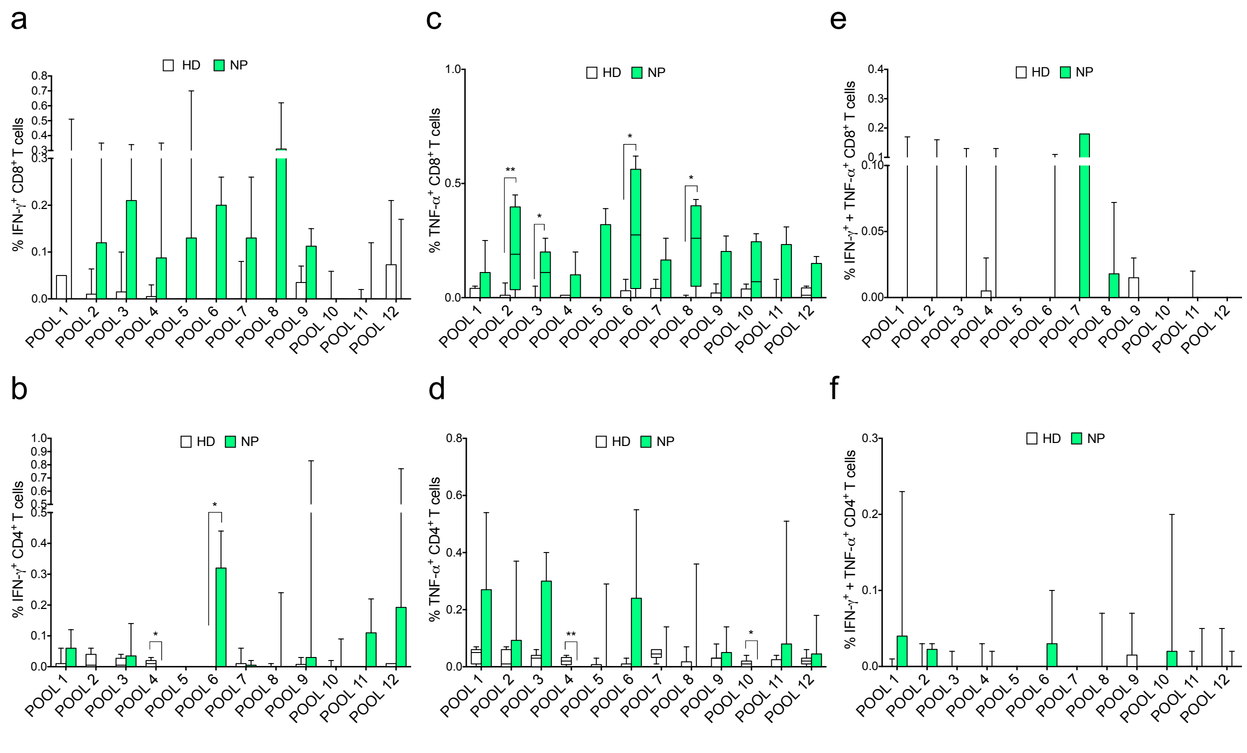

Supplementary Figure 6

Supplementary Figure 6. CD8⁺ and CD4⁺ Teff cell responses against NM-neoAg epitopes from apoptotic NSCLC cells in naïve NSCLC patients. Analysis of IFN-γ (a and b), TNF-α (c and d) and IFN-γ + TNF-α (e and f) production respectively by CD8⁺ and CD4⁺ T cells in response to 12 pools with overlapping NM-neoAg peptides. Comparison between cytokine production in healthy donors (HD; n=10), and naive NSCLC patients who did not require neo-adjuvant chemotherapy, and whose blood sample was obtained immediately before (1 day) the surgery resection (NP=naïve patients; n=7). All values were subtracted from background. Bars were showed as box and whisker graphs. *$p < 0.05$; **$p < 0.01$ by unpaired Student's *t*-test.

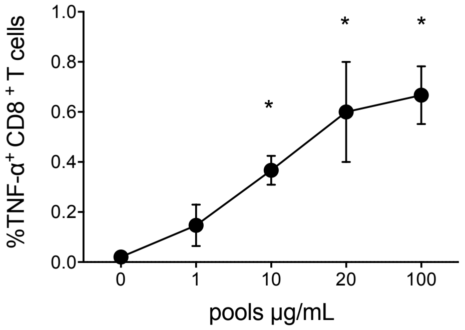

Supplementary Figure 7

Supplementary Figure 7. Dose-response curve of NM-neoAg-specific CD8^{+} T cells. The graph shows a dose response curve of TNF-α production following PBMC stimulation with the indicated concentrations of peptide pool 2 (the background [BG] was subtracted), as detected by FC analysis; *$p < 0.05$ by multiple *t* test with Holm-Sidak method.

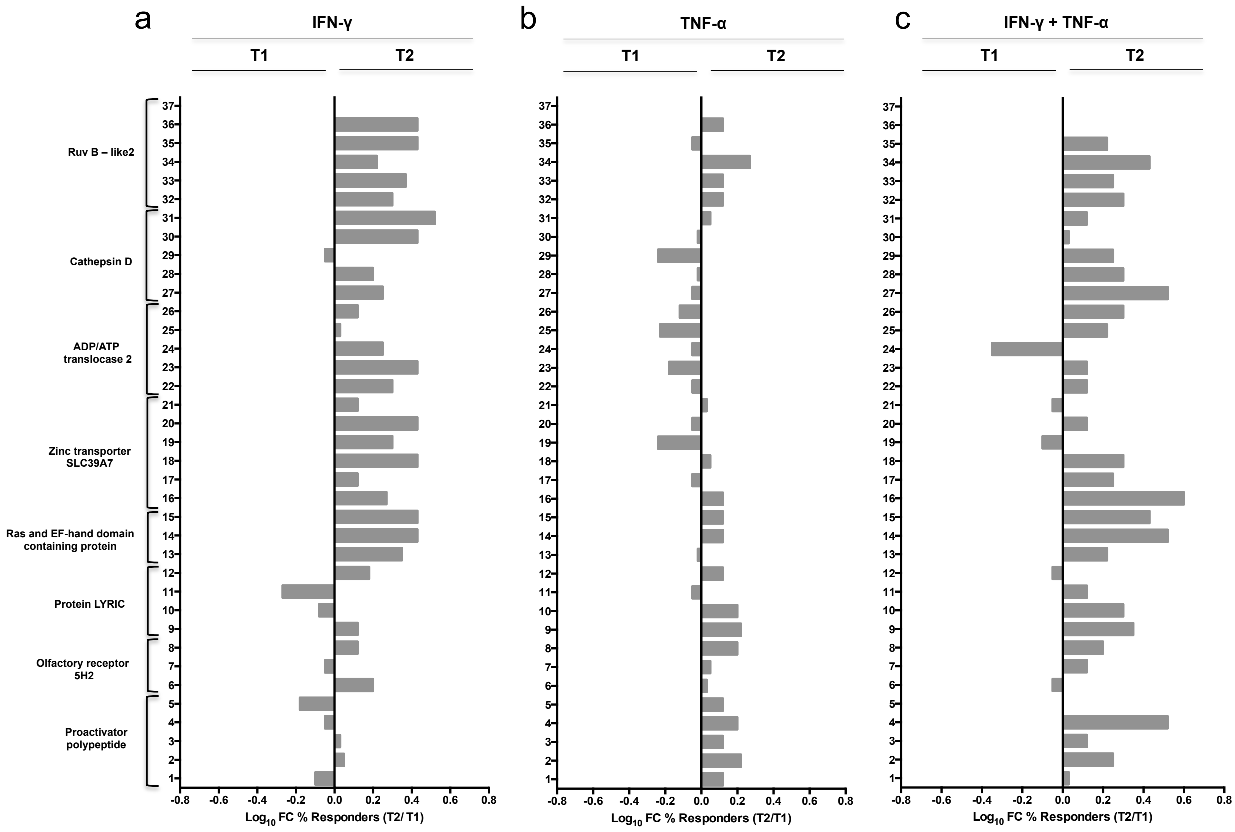

Supplementary Figure 8

Supplementary Figure 8. Comparison between percentage of CDDP- and CDDP&Nivolumab-treated patients with CD8⁺ Teff cells responding to the single peptides. (a-c) Fold change [FC], calculated as ratio of percentage of patients studied at the T2 (n=12, after the subsequent nivolumab treatment) on the percentage of patients studied at the T1 (n=14, after the chemotherapy protocol including CDDP) for the $CD8^+$ Teff cells producing IFN-γ (a), TNF-α (b), or both (c) in response to the single peptides (FC, calculated as ratio of T2/T1).

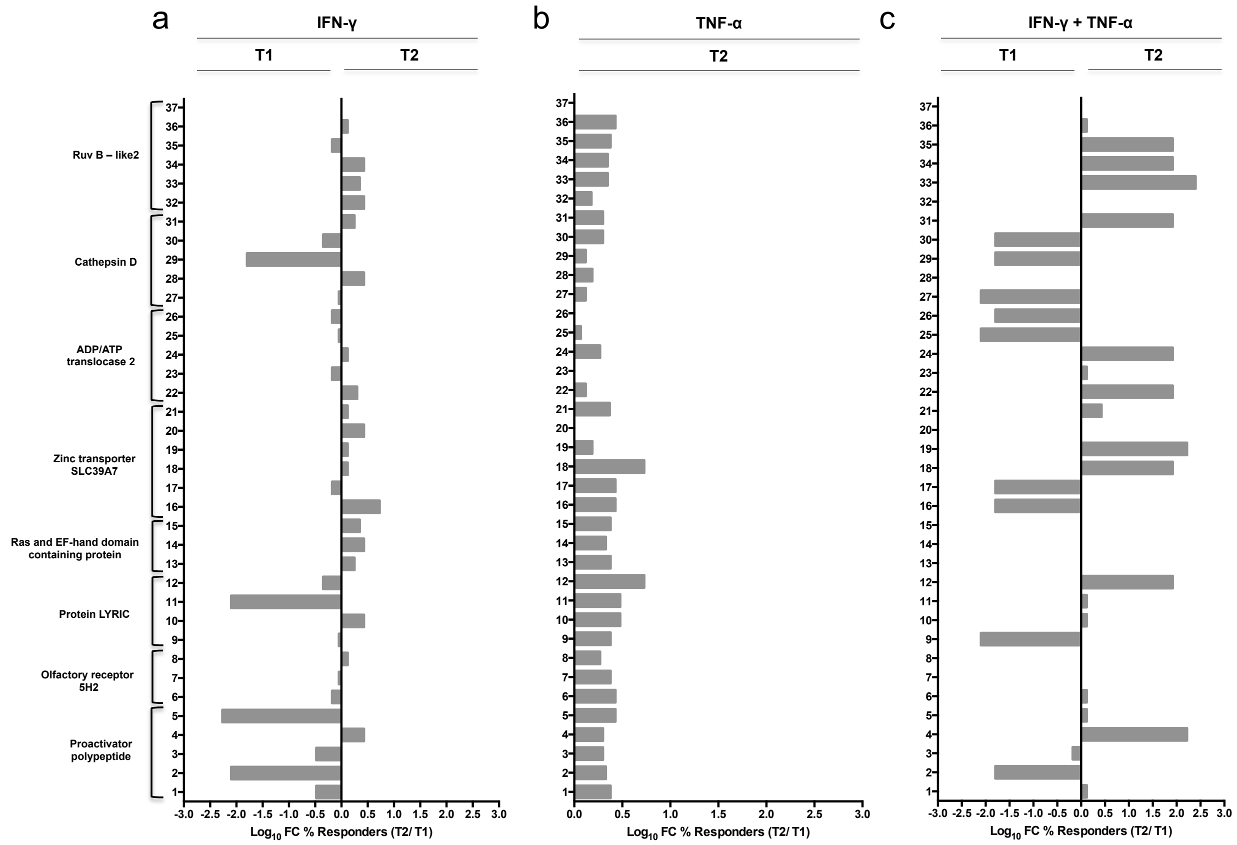

Supplementary Figure 9

Supplementary Figure 9. Comparison between percentage of CDDP- and CDDP&Nivolumab-treated patients with CD4⁺ Teff cells responding to the single peptides. (a, c) Fold change [FC], calculated as ratio of percentage of patients studied at the T2 (n=12, after the subsequent nivolumab treatment) on the percentage of patients studied at the T1 (n=14, after the chemotherapy protocol including CDDP) for the CD4⁺ Teff cells producing IFN-γ (a), TNF-α (b), or both (c) in response to the single peptides (FC, calculated as ratio of T2/T1).

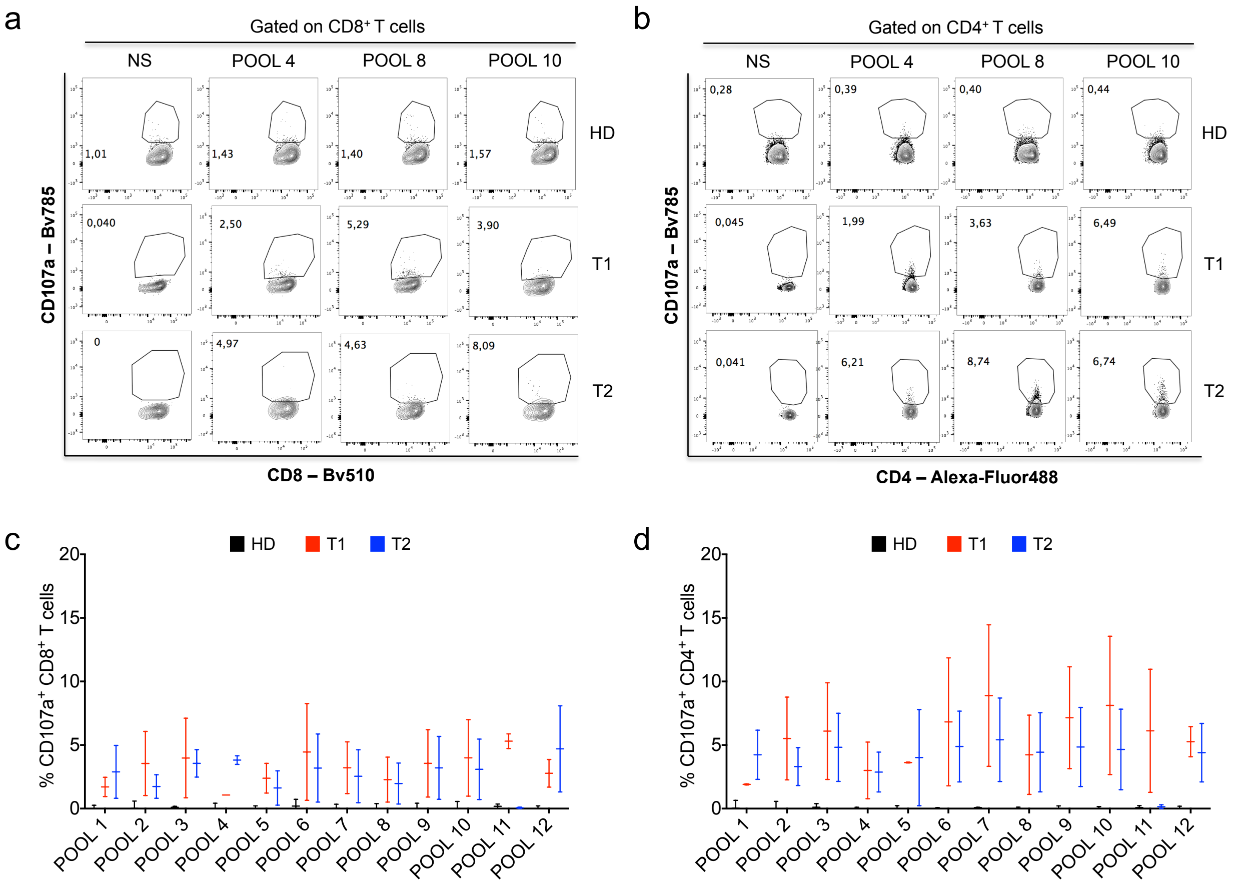

Supplementary Figure 10

Supplementary Figure 10. CD107a upregulation by CD4^{+} and CD8^{+} T cells in response to NSCLC-associated NM-neoAg epitopes. (a, b) Representative contour plot FC analysis of CD107a^{+} cells in CD8^{+} T cells (a) and CD4^{+} T cells (b) from a HD, a CDDP-treated patient (T1) or a CDDP&Nivolumab-treated patient (T2), after stimulation or not with 20 µg/mL of peptide pools. (c, d) Percentage of CD107a^{+} cells in CD8^{+} T cells (c) and CD4^{+} T cells (d) from HDs (n=3), T1 (n=2) or T2 (n=2), upon stimulation with 20 µg/mL of peptide pools (background was subtracted). Bars were showed as box and whisker graphs.

a

Protein Name	Peptide Number	Sequence
GLYCERALDEHYDE-3 PHOSPHATE DEHYDROGENASE	1	GVFTTMEKAGAHLQGGAKRV
	2	AGAHLQGGAKRVIISAPSAD
	3	AKRVIISAPSADAPMFVMGV
	4	PSADAPMFVMGVNHEKYDNS
	5	VMGVNHEKYDNSLKIISNAS
	6	YDNSLKIISNASCTTNCLAP
	7	SNASCTTNCLAPLAKVIHDN
	8	CLAPLAKVIHDNFGIVEGLM
	9	IHDNFGIVEGLMTTVHAITA
	10	EGLMTTVHAITATQKTVDGP
	11	AITATQKTVDGPSGKLWRDG
	12	VDGPSGKLWRDGRGALQNII
TUBULIN BETA-4B CHAIN	1	YCIDNEALYDICFRTLKLTT
	2	YDICFRTLKLTTPTYGDLNH
	3	KLTTPTYGDLNHLVSATMSG
	4	DLNHLVSATMSGVTTCLRFP
	5	TMSGVTTCLRFPGQLNADLR
HETEROGENEOUS NUCLEAR RIBONUCLEOPROTEIN A1	1	FVTFDDHDSVDKIVIQKYHT
	2	SVDKIVIQKYHTVNGHNCEV
	3	KYHTVNGHNCEVRKALSKQE
	4	NCEVRKALSKQEMASASSSQ
	5	SKQEMASASSSQRGRSGSGN

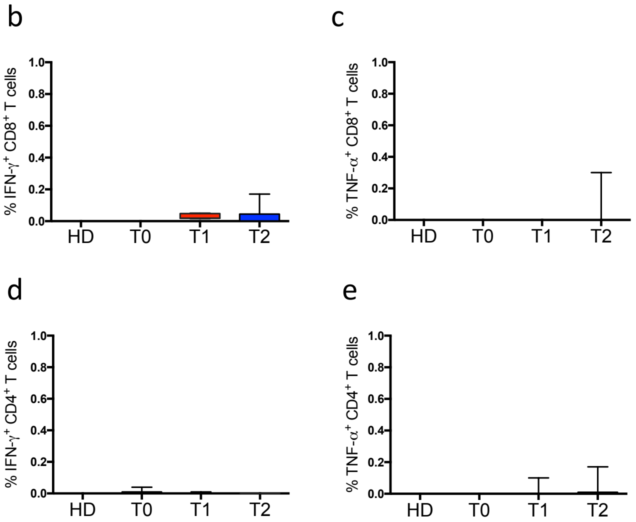

Supplementary Figure 11

Supplementary Figure 11. T cells responses after stimulation with control proteins (up-regulated in viable cells). (a) 22 peptides derived from three down-regulated proteins (Glyceraldehyde-3 phosphate dehydrogenase, Tubulin beta-4B chain and Heterogeneous nuclear ribonucleoprotein A1) in NSCLC apoptotic fraction were used as control. Each peptide shows a 20 amino acid sequence with overlapping region of 12 residues. A sequential number was assigned for each peptide and a distinct color code was assigned for each protein. Protein name and relative amino acid sequences were showed. (b-e) Mean values of IFN-γ and TNF-α production by $CD8^+$ (b and c) or $CD4^+$ T cells (d and e) in response to a pool of 22 peptides were showed. Comparison between healthy donors (HD; n=10); patients before (T0; n=6) and after CDDP administration (T1; n=14) and patients who, after CDDP, were submitted to Nivolumab treatment (T2; n=12). Bars were showed as box and whisker graphs.

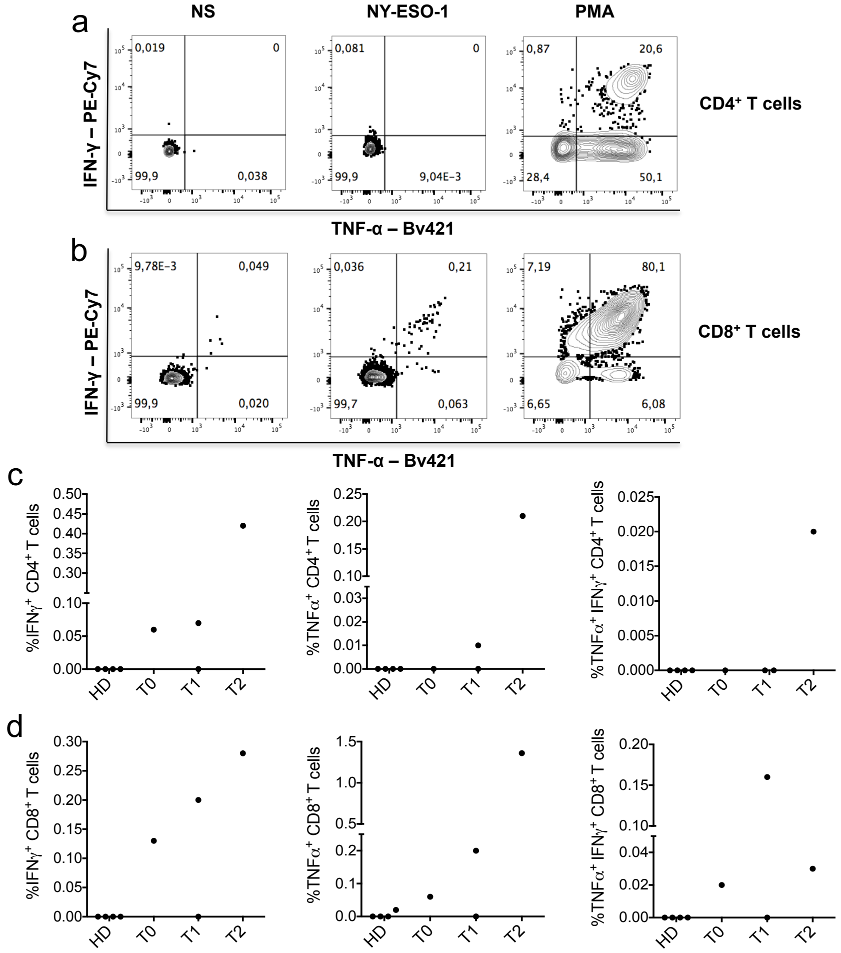

Supplementary Figure 12

Supplementary Figure 12. Teff cell responses to NY-ESO1. Representative FC (contour plot) analysis of cytokine production (IFN-γ and TNF-α) by $CD4^+$ Teff cells (a) or $CD8^+$ Teff cells (b) in response or not (NS) to Peptivator NY-ESO-1-premium grade in a patient after CDDP-chemotherapy (T1). A positive control (PMA) was included. Percentage of $CD4^+$ Teff cells (c) or $CD8^+$ Teff cells (d) producing IFN-γ, TNF-α or both, in response to Peptivator NY-ESO-1-premium grade. The FC analyses were performed in PBMCs obtained from 4 HDs and NSCLC patients before any treatment (T0 N=1), after CDDP-chemotherapy (T1 N=2), and after nivolumab therapy (T2 N=1). Data were showed as dot plots.

Supplementary Tables

Supplementary Table 1. *Demographic and clinical characteristics of NSCLC patients who experienced surgery treatment without neoadjuvant therapy*

	All
Number of patients	7
Gender M/F	3/4
Age (mean±SD); years range	68±9; 58-79
Tumor histology:<br>*LUAD*	 7/7
Tumor stage:<br>*IA*<br>*IIA*<br>*IIIA*<br>*IIIB*	 1/7<br>1/7<br>4/7<br>1/7
Mutations:<br>*EGFR*<br>*ALK*<br>*KRAS*	 1/7<br>0/7<br>2/7

LUAD: lung adenocarcinoma; **EGFR**: epidermal growth factor receptor; **ALK**: anaplastic lymphoma kinase; **KRAS**: kirsten rat sarcoma virus.

Supplementary Table 2. *List of Abs used in flow cytometry and immunohistochemistry analyses*

Marker	Fluorochrome	Clone	Company	Catalog
Viability dye	eFluor780	–	eBioscience	65-0865-18
CD8	BV-510	SK1	Biolegend	344732
CD45RA	BV-605	HI100	Biolegend	304133
CCR7	PerCP-Cy5.5	G043H7	Biolegend	353220
PD-1	APC	EH12.2H7	Biolegend	329908
CD107a	BV-785	H4A3	BioLegend	328644
IFN-Y	PE-Cy7	4S.B3	BioLegend	502528
TNF-α	BV-421	MAb11	BioLegend	502932
CD4	Alexa-Fluor488	OKT4	eBioscience	53-0048-42
CD14	APC-eFluor780	61D3	eBioscience	47-0149-42
CD16	APC-eFluor780	CB16	eBioscience	47-0168-42
CD56	APC-eFluor780	CMSSB	eBioscience	47-0567-42
CD19	APC-eFluor780	HIB19	eBioscience	47-0199-42
Annexin V	FITC		BioLegend	640906
Act Caspase 3	V450	C92-605	BD	560627
EpCAM (CD326)	FITC	HEA-125	Miltenyi	130-080-301
PSAP*	–	4D5F4	Abcam	Ab189425
LYRIC*	–	Polyclonal	Abcam	Ab76742
Cleaved Caspase-3*	–	Polyclonal	Cell Signaling	9661

*Used in immunohistochemistry analysis at 1:200 dilution.

All antibodies used in flow cytometry analyses were used at 1:50 dilution, except for CD14, CD16, CD56, CD19 (1:100) and CD107a (1:200).

Supplementary Notes

Relevant data on tumor-associated proteins found upregulated in CDDP-ap NSCLC cells in form of fragmented proteins.

1. **AFG3-like protein 2*: it is upregulated in several cancers including lung cancers (https://www.proteinatlas.org/ENSG00000141385-AFG3L2/pathology).

2. **CAAX prenyl protease 1 homolog*: no data found in tumors.

3. **Putative elongation factor 1-alpha-like 3*: its overexpression predicted poor prognosis in various tumors including lung cancer [2].

4. **Reticulon-3*: The reticulons are a group of highly conserved genes with preferential expression in neuroendocrine tissues; no data found om lung tumors. (https://www.ncbi.nlm.nih.gov/gene?Db=gene&Cmd=ShowDetailView&TermToSearch=10313); reticulon-3 is upregulated in several cancers including lung cancers (https://www.proteinatlas.org/ENSG00000133318-RTN3/pathology).

5. **Reticulon-4*: The reticulons are a group of highly conserved genes with preferential expression in neuroendocrine tissues (https://www.ncbi.nlm.nih.gov/gene?Db=gene&Cmd=ShowDetailView&TermToSearch=10313); reticulon-4 is upregulated in several cancers including lung cancers (https://www.proteinatlas.org/ENSG00000115310-RTN4/pathology).

6. **zinc transporter SLC39A7*: it plays crucial role in growth of colorectal cancer [3], and is overexpressed in NSCLC [4];

7. **Beta-hexosaminidase subunit beta*: it is upregulated in several cancers including lung cancers (https://www.proteinatlas.org/ENSG00000049860-HEXB/pathology).

8. **Mitochondrial inner membrane protein*: it is highly expressed in tumor brains [5]; no data found on lung tumors.

9. **ADP/ATP translocase 2*: it is a mitochondrial carrier, transcriptional target of p53 and overexpressed in NSCLC as a negative prognostic marker [6];

10. **chatepsin D*: it is overexpressed in lung cancers [7];

11. ***ADP/ATP translocase 3*: it is a regulatory component of the mitochondrial permeability controlling controls mitochondrial permeability transition during apoptosis [8]; no data found om lung tumors.

12. ***ruvB-like 2: it belongs to AAA+ ATPase family*: it is required for the transforming activity of c-myc [9], beta-catenin[10], and the viral oncoprotein E1A [11] and it is upregulated in several tumors including liver [12], colon [13, 14], lymphoma [15], NSCLC [16].

13. ***OCIA domain-containing protein 1*: cancer-related protein related with malignancy of ovarian mucinous tumors [17]; no data found in lung tumors. (https://www.proteinatlas.org/ENSG00000184009-ACTG1/pathology; Cadinu D et al EuPA Open Proteomics, 2014).

14. ***Tubulin alpha-1C chain*: it is is upregulated in several cancers including lung cancers (https://www.proteinatlas.org/ENSG00000167553-TUBA1C/pathology); it has been suggested to promote migration and proliferation and to predict a poor prognosis in hepatocellular carcinoma [18], breast cancer [19].

15. ***Vesicle-associated membrane protein-associated protein A*: is involved in vesicle trafficking; no data found on lung tumors.

16. ****dipeptidyl peptidase 1*: no data found in tumors.

17. ****Actin, cytoplasmic 2*: it is upregulated in several cancers including lung cancers

18. ****olfactory receptor 5H2*: it is overexpressed by several tumors including NSCLC [20];

19. ****Ras and EF-hand domain containing protein*: it plays a significant role in lung cancer cell-growth [21];

20. ****proactivator polypeptide*: it is upregulated in some cases of various cancers including NSCLC (https://www.proteinatlas.org/)];

21. ****protein LYRIC*: it acts as an oncogene in melanoma, breast cancer or hepatocellular carcinoma [22], and is upregulated in various types of tumors including NSCLC [23].

* Proteins only found by SILAC forward

** Proteins only found by SILAC Reverse

*** Proteins found by both SILAC forward and reverse

Supplementary References

1. Mancone C*, et al.* Iron overload down-regulates the expression of the HIV-1 Rev cofactor eIF5A in infected T lymphocytes. *Proteome science* **15**, 18 (2017).

2. Hassan MK, Kumar D, Naik M, Dixit M. The expression profile and prognostic significance of eukaryotic translation elongation factors in different cancers. *PloS one* **13**, e0191377 (2018).

3. Sheng N*, et al.* Knockdown of SLC39A7 inhibits cell growth and induces apoptosis in human colorectal cancer cells. *Acta biochimica et biophysica Sinica* **49**, 926-934 (2017).

4. Huang C, Cui X, Sun X, Yang J, Li M. Zinc transporters are differentially expressed in human non-small cell lung cancer. *Oncotarget* **7**, 66935-66943 (2016).

5. Zhao J*, et al.* The novel conserved mitochondrial inner-membrane protein MTGM regulates mitochondrial morphology and cell proliferation. *Journal of cell science* **122**, 2252-2262 (2009).

6. Kolukula VK*, et al.* SLC25A1, or CIC, is a novel transcriptional target of mutant p53 and a negative tumor prognostic marker. *Oncotarget* **5**, 1212-1225 (2014).

7. Lou X*, et al.* Cathepsin D is secreted from M-BE cells: its potential role as a biomarker of lung cancer. *Journal of proteome research* **6**, 1083-1092 (2007).

8. Yang Z*, et al.* Adenine nucleotide (ADP/ATP) translocase 3 participates in the tumor necrosis factor induced apoptosis of MCF-7 cells. *Molecular biology of the cell* **18**, 4681-4689 (2007).

9. Wood MA, McMahon SB, Cole MD. An ATPase/helicase complex is an essential cofactor for oncogenic transformation by c-Myc. *Molecular cell* **5**, 321-330 (2000).

10. Feng Y, Lee N, Fearon ER. TIP49 regulates beta-catenin-mediated neoplastic transformation and T-cell factor target gene induction via effects on chromatin remodeling. *Cancer research* **63**, 8726-8734 (2003).

11. Dugan KA, Wood MA, Cole MD. TIP49, but not TRRAP, modulates c-Myc and E2F1 dependent apoptosis. *Oncogene* **21**, 5835-5843 (2002).

12. Li C*, et al.* Proteomic analysis of hepatitis B virus-associated hepatocellular carcinoma: Identification of potential tumor markers. *Proteomics* **5**, 1125-1139 (2005).

13. Carlson ML, Wilson ET, Prescott SM. Regulation of COX-2 transcription in a colon cancer cell line by Pontin52/TIP49a. *Molecular cancer* **2**, 42 (2003).

14. Lauscher JC, Loddenkemper C, Kosel L, Grone J, Buhr HJ, Huber O. Increased pontin expression in human colorectal cancer tissue. *Human pathology* **38**, 978-985 (2007).

15. Nishiu M, *et al.* Microarray analysis of gene-expression profiles in diffuse large B-cell lymphoma: identification of genes related to disease progression. *Japanese journal of cancer research : Gann* **93**, 894-901 (2002).

16. Dehan E, *et al.* Chromosomal aberrations and gene expression profiles in non-small cell lung cancer. *Lung cancer* **56**, 175-184 (2007).

17. Wang C, Michener CM, Belinson JL, Vaziri S, Ganapathi R, Sengupta S. Role of the 18:1 lysophosphatidic acid-ovarian cancer immunoreactive antigen domain containing 1 (OCIAD1)-integrin axis in generating late-stage ovarian cancer. *Molecular cancer therapeutics* **9**, 1709-1718 (2010).

18. Wang J, Chen W, Wei W, Lou J. Oncogene TUBA1C promotes migration and proliferation in hepatocellular carcinoma and predicts a poor prognosis. *Oncotarget* **8**, 96215-96224 (2017).

19. Boggs AE, *et al.* alpha-Tubulin acetylation elevated in metastatic and basal-like breast cancer cells promotes microtentacle formation, adhesion, and invasive migration. *Cancer research* **75**, 203-215 (2015).

20. Ranzani M, *et al.* Revisiting olfactory receptors as putative drivers of cancer. *Wellcome open research* **2**, 9 (2017).

21. Oshita H, *et al.* RASEF is a novel diagnostic biomarker and a therapeutic target for lung cancer. *Molecular cancer research : MCR* **11**, 937-951 (2013).

22. Yoo BK, *et al.* Astrocyte elevated gene-1 (AEG-1): A multifunctional regulator of normal and abnormal physiology. *Pharmacology & therapeutics* **130**, 1-8 (2011).

23. Yao Y, *et al.* Metadherin regulates proliferation and metastasis via actin cytoskeletal remodelling in non-small cell lung cancer. *British journal of cancer* **111**, 355-364 (2014).
