# Supplementary material for: Combination of chemotherapy and PD-1 blockade induces T cell responses to tumor non-mutated neoantigens
Source: Commun Biol. 2020 Feb 25;3:85. doi: 10.1038/s42003-020-0811-x (PMC7042341; doi:10.1038/s42003-020-0811-x)
Supplement: Supplementary file 2 — Description of Additional Supplementary Files [file 42003_2020_811_MOESM2_ESM.pdf]

Description of additional supplementary items

Supplementary Data 1. *List of proteins identified and quantified in primary NSCLC cell line by SILAC (Forward and Reverse)-based MS. (see the related Excel file in Supplementary Data 1).* Worksheet SILAC Forward: list of 815 identified proteins. ProteinPilot Unused score, ProteinPilot Total score, % of Coverage sequence, UniprotKD Accession Number, Protein Name, Unique Peptides (95%) and Fold change of SILAC Forward Ratio (Heavy apoptotic cells / Light live cells) are showed for each protein. 16 proteins identified as upregulated fragments in apoptotic cells are highlighted in yellow.
Worksheet SILAC Reverse - Matched proteins: list of 369 matched proteins identified in both forward and reverse SILAC experiment. ProteinPilot Unused score, ProteinPilot Total score, % of Coverage sequence, UniprotKD Accession Number, Protein Name, Unique Peptides (95%), Fold change of SILAC Forward Ratio (Heavy apoptotic cells / Light live cells) and Fold change of SILAC Reverse Ratio (Light apoptotic cells / Heavy live cells) are showed for each protein. 6 matched proteins identified as upregulated fragments in apoptotic cells are highlighted in yellow.

Supplementary Data 2. *Source data presented in the main figures. (see the related Excel file in Supplementary Data 2).* Each worksheet contains raw data of related figure (figures 2-7).
