# Supplementary material for: Combination of chemotherapy and PD-1 blockade induces T cell responses to tumor non-mutated neoantigens
Source: Commun Biol. 2020 Feb 25;3:85. doi: 10.1038/s42003-020-0811-x (PMC7042341; doi:10.1038/s42003-020-0811-x)
Supplement: Supplementary file 5 — Reporting Summary [file 42003_2020_811_MOESM5_ESM.pdf]

nature research

Corresponding author(s): Vincenzo Barnaba

Last updated by author(s): Dec 30, 2019

Reporting Summary

Nature Research wishes to improve the reproducibility of the work that we publish. This form provides structure for consistency and transparency in reporting. For further information on Nature Research policies, see Authors & Referees and the Editorial Policy Checklist.

Statistics

For all statistical analyses, confirm that the following items are present in the figure legend, table legend, main text, or Methods section.

n/a	Confirmed	
☐	☒	The exact sample size (n) for each experimental group/condition, given as a discrete number and unit of measurement
☐	☒	A statement on whether measurements were taken from distinct samples or whether the same sample was measured repeatedly
☐	☒	The statistical test(s) used AND whether they are one- or two-sided<br>*Only common tests should be described solely by name; describe more complex techniques in the Methods section.*
☒	☐	A description of all covariates tested
☐	☒	A description of any assumptions or corrections, such as tests of normality and adjustment for multiple comparisons
☐	☒	A full description of the statistical parameters including central tendency (e.g. means) or other basic estimates (e.g. regression coefficient) AND variation (e.g. standard deviation) or associated estimates of uncertainty (e.g. confidence intervals)
☐	☒	For null hypothesis testing, the test statistic (e.g. F, t, r) with confidence intervals, effect sizes, degrees of freedom and P value noted<br>*Give P values as exact values whenever suitable.*
☒	☐	For Bayesian analysis, information on the choice of priors and Markov chain Monte Carlo settings
☒	☐	For hierarchical and complex designs, identification of the appropriate level for tests and full reporting of outcomes
☒	☐	Estimates of effect sizes (e.g. Cohen's d, Pearson's r), indicating how they were calculated

Our web collection on statistics for biologists contains articles on many of the points above.

Software and code

Policy information about availability of computer code

Data collection	4000 series explorer software version 4.1.0<br>ProteinPilot Software 4.5<br>MASCOT search engine Version 2.1<br>FACSDiva software version 6.1.3<br>FlowJo version 10.0
Data analysis	Graph Pad Prism version 6.0<br>Panther classification system version 14.1-2018_04

For manuscripts utilizing custom algorithms or software that are central to the research but not yet described in published literature, software must be made available to editors/reviewers. We strongly encourage code deposition in a community repository (e.g. GitHub). See the Nature Research guidelines for submitting code & software for further information.

Data

Policy information about availability of data

All manuscripts must include a data availability statement. This statement should provide the following information, where applicable:

- Accession codes, unique identifiers, or web links for publicly available datasets
- A list of figures that have associated raw data
- A description of any restrictions on data availability

There are no restrictions on data availability and will be made available upon request.

Field-specific reporting

Please select the one below that is the best fit for your research. If you are not sure, read the appropriate sections before making your selection.

☒ Life sciences ☐ Behavioural & social sciences ☐ Ecological, evolutionary & environmental sciences

For a reference copy of the document with all sections, see nature.com/documents/nr-reporting-summary-flat.pdf

Life sciences study design

All studies must disclose on these points even when the disclosure is negative.

Sample size	No sample size calculation was performed
Data exclusions	No data ware excluded
Replication	Proteomic analysis was repeated 2 times performing a forward and reverse SILAC replicate obtaining similar results. Cells number obtained from human PBMCs allowed us to perform a single replicate for immunological validation.<br>Flow cytometry analysis was performed with blood samples collected from NSCLC patients at different time point of therapy. When PBMCs amount was enough, the reproducibility of FC assay was confirmed by duplicate experiments.
Randomization	Sample randomization is not applicable to the present study
Blinding	Not applicable

Reporting for specific materials, systems and methods

We require information from authors about some types of materials, experimental systems and methods used in many studies. Here, indicate whether each material, system or method listed is relevant to your study. If you are not sure if a list item applies to your research, read the appropriate section before selecting a response.

Materials & experimental systems

n/a	Involved in the study	
☐	☒	Antibodies
☐	☒	Eukaryotic cell lines
☒	☐	Palaeontology
☒	☐	Animals and other organisms
☐	☒	Human research participants
☒	☐	Clinical data

Methods

n/a	Involved in the study	
☒	☐	ChIP-seq
☐	☒	Flow cytometry
☒	☐	MRI-based neuroimaging

Antibodies

Antibodies used

Antibodies for flow cytometry (FC) or immunohistochemistry (IHC) were purchased from eBioscience, Biolegend, BD, Myltenyi, Abcam and Cell Signaling. For IHC anitbodies were used at a dilution of 1:200. For FC anitbodies were used at a dilution of 1:50, except for CD14, CD16, CD56, CD19 (1:100) and CD107a (1:200).
Human antibodies for FC:
Viability dye eFluor780 eBioscience 65-0865-18
CD8 BV-510 SK1 Biolegend 344732
CD45RA BV-605 HI100 Biolegend 304133
CCR7 PerCP-Cy5.5 G043H7 Biolegend 353220
PD-1 APC EH12.2H7 Biolegend 329908
CD107a BV-785 H4A3 BioLegend 328644
IFN-Y PE-Cy7 4S.B3 BioLegend 502528
TNF-α BV-421 MAb11 BioLegend 502932
CD4 Alexa-Fluor488 OKT4 eBioscience 53-0048-42
CD14 APC-eFluor780 61D3 eBioscience 47-0149-42
CD16 APC-eFluor780 CB16 eBioscience 47-0168-42
CD56 APC-eFluor780 CMSSB eBioscience 47-0567-42
CD19 APC-eFluor780 HIB19 eBioscience 47-0199-42
Annexin V FITC BioLegend 640906
Act Caspase 3 V450 C92-605 BD 560627
EpCAM (CD326) FITC HEA-125 Miltenyi 130-080-301
Human antibodies for IHC:
PSAP 4D5F4 Abcam Ab189425
LYRIC Polyclonal Abcam Ab76742

	Cleaved Caspase-3 Polyclonal Cell Signaling 9661
Validation	All antibodies were validated by the manufacturer, please refer to the catalogue information above validation with respective vendors.

Eukaryotic cell lines

Policy information about cell lines

Cell line source(s)	Primary NSCLC cell line obtained from a human adenocarcinoma tissue
Authentication	No specific cell line authentication was performed
Mycoplasma contamination	Test for mycoplasma contamination was performed before and during experiments with primary NSCLC cell line
Commonly misidentified lines (See ICLAC register)	Primary NSCLC cell line used is not listed in the ICLAC database

Human research participants

Policy information about studies involving human research participants

Population characteristics	Human studies were performed in accordance with the ethical guidelines of the 1975 Declaration of Helsinki and approved by the Institutional Ethical Committee. Informed consent was obtained from healthy subjects and all patients. Healthy human blood samples were obtained from blood center of Policlinico Umberto I - Rome. Healthy human subjects were sex matched male and female. Blood samples from NSCLC patients were obtained from Oncology Center of Sapienza University of Rome and Regina Elena National Cancer Institute in Rome. Detailed information on the patient population in cluded in the study is provided in Methods and Supplementary Data.
Recruitment	Study partecipants were selected to form a prospective observational study at the .Sapienza University or Rome. Histological diagnosis was determined based on microscopic features of carcinoma cells. We enrolled and monitored 14 NSCLC IV stage patients: all patients were studied before and after various cycles of a chemotherapy protocol, whereas 12 of them were also studied after a subsequent treatment with several cycles with nivolumab. Nivolumab treatment started after failure (demonstrated by instrumental and/or biochemical metastatic progression) of chemotherapy. Demographic, clinical, and therapeutic characteristics of these NSCLC patients are described in Table 2.<br>Seven patients with earlier NSCLC stage were also included in this study. These patients did not require neo-adjuvant chemotherapy, and whose blood sample was obtained immediately before (1 day) the surgery resection. Demographic, clinical, and therapeutic characteristics of these NSCLC patients are described in Supplementary Table 2.
Ethics oversight	Human studies were approved by the Institutional Ethical Committee of Policlinico Umberto I - Sapienza University of Rome

Note that full information on the approval of the study protocol must also be provided in the manuscript.

Flow Cytometry

Plots

Confirm that:

- [x] The axis labels state the marker and fluorochrome used (e.g. CD4-FITC).
- [x] The axis scales are clearly visible. Include numbers along axes only for bottom left plot of group (a 'group' is an analysis of identical markers).
- [x] All plots are contour plots with outliers or pseudocolor plots.
- [x] A numerical value for number of cells or percentage (with statistics) is provided.

Methodology

Sample preparation	PBMCs from NSCLC patients and HDs<br>PBMCs were isolated from fresh heparinized blood by density gradient centrifugation with Lympholyte and collected in complete RPMI medium containing 10% heat-inactivated FBS. Cells were stimulated or not with 20 µg/mL peptide pool plus 1 µg/mL of anti-CD28 mAb and the Protein Transport Inhibitor Cocktail or with the Cell Stimulation plus Protein Transport Inhibitor Cocktail as a positive control, and mAb to CD107a for degranulation analysis, for 18 hours at 37°C. After antigen stimulation, cells were washed and stained with Fixable viability Dye eFluor780 for the exclusion of dead cells, in PBS 30 minutes at room temperature. After washing, surface staining was performed incubating cells with the labelled mAbs to CD4, CD8, CCR7, CD45RA, PD-1 and with a cocktail of labelled mAbs to CD14, CD16, CD56, CD19 (dump channel was included for the exclusion of monocytes, NK cells, and B cells, respectively) for 20 min at 4°C, in PBS containing 2% FBS. To analyse cytokines production, cells were fixed and permeabilized using the BD Cytofix/Cytoperm Fixation/Permeabilization Solution Kit at 4°C for 20 minutes, washed, and stained with mAbs to IFN-γ and TNF-α for 20 minutes at 4°C, in BD Perm/Wash buffer
Instrument	Cells were acquired with LSRFortessa cytometer (BD Biosciences)

Software	Data were acquired with FACSDiva software version 6.1.3 and with FlowJo software version 10.0 (Tree Star)
Cell population abundance	Cell populations were FACS sorted to >95% purity
Gating strategy	For all relevant experiments with PBMCs, lymphocytes were first gated by the physical parameter Forward and Side scatter area (FSC-A and SSC-A) and doublets and debris were excluded by plotting the width (W) against the area of FSC and SSC parameters. Dead cells were excluded using viability dye (VD), and gating into live cells we identified CD8+ and CD4+ T cells. A dump channel was included for exclusion of B cells, monocytes and NK cells (CD19, CD14, CD16, CD56).<br>In CD8+ and CD4+ T cells populations we identified: subsets of interest (N,CM,EM,EMRA), cytokine production (IFN-γ, TNF-α), degranulation marker (CD107a) and PD1 expression. In some staining we used the FMO (fluorescences minus one) to discriminate the positive and negative populations.

☒ Tick this box to confirm that a figure exemplifying the gating strategy is provided in the Supplementary Information.
